# Erector Spinae Plane Block With Liposomal Bupivacaine for Cervical Spine Surgery

**DOI:** 10.1155/cria/5687388

**Published:** 2025-12-09

**Authors:** Hali Peterson, Ahmad Nassr, Mitchell Kerfeld

**Affiliations:** ^1^ Department of Anesthesiology and Perioperative Medicine, Mayo Clinic, Rochester, Minnesota, USA, mayo.edu; ^2^ Department of Orthopedic Surgery, Mayo Clinic, Rochester, Minnesota, USA, mayo.edu

## Abstract

Regional anesthesia for spine surgery is a relatively new and evolving area. Erector spinae plane blocks (ESPB) are a type of regional technique that has successfully managed acute postoperative pain in thoracic and lumbar spine surgeries. Using ESPB in cervical spine surgeries has shown to reduce intraoperative opioid needs and improve postoperative pain control, among other benefits. Liposomal bupivacaine has been shown potential in managing acute postoperative pain when injected at the surgical incision site. There is limited research on using liposomal bupivacaine in cervical spine surgery, especially with ESPB. We present a case of ESPB for cervical spine surgery performed successfully with liposomal bupivacaine.

## 1. Introduction

Cervical spine surgery presents a challenge to the anesthesiologist for perioperative pain management and requires a multimodal approach to effectively alleviate postoperative discomfort and improve patient outcomes. Research has demonstrated that erector spinae plane blocks (ESPB) can facilitate pain relief following cervical spine surgery; however, their efficacy may depend on the incorporation of additives to prolong the duration of postoperative pain relief. Liposomal bupivacaine, noted for its extended analgesic effects, has demonstrated potential in the management of acute postoperative pain following spine surgery when infiltrated at the surgical incision site [1]. Currently, there is limited research on the use of liposomal bupivacaine in cervical spine surgery, specifically with ESPB. Currently, Exparel is FDA approved for interscalene, sciatic, popliteal fossa, and adductor canal nerve blocks, as well as local infiltration. Most existing research on ESPB in spine surgery has primarily focused on its application in surgeries on the lumbar spine [2, 3]. The incorporation of liposomal bupivacaine into ESPB may represent a synergistic approach to enhancing pain control following cervical spine surgery.

## 2. Case Presentation

A 66‐year‐old male with cervical myeloradiculopathy presented for posterior cervical laminoplasty and decompression of levels C3 through C7, including right foraminotomies at C4‐5, C5‐6, and C6‐7. The patient was classified as American Society of Anesthesiologists physical status III, with medical comorbidities such as hypertension, Type 2 diabetes mellitus, hypothyroidism, and nicotine dependence. He had experienced neck pain accompanied by bilateral upper extremity numbness, tingling, and electric shock‐like sensations for several years and had been taking gabapentin. Electromyography revealed evidence of chronic left C8 and right C5 through C7 radiculopathies.

The anesthesia team planned bilateral single‐injection ESPB at the T1 level. Preoperatively, he received 1000 mg of oral acetaminophen. Procedural sedation for ESPB consisted of 50 mcg of intravenous fentanyl and 2 mg of intravenous midazolam. The ESPB was performed using a SonoSite C60xp/5‐2 curvilinear probe. In the sitting position, the posterior part of the shaft of the first rib was identified under ultrasound guidance and traced medially to locate the transverse process of T1. A 20G echogenic needle was inserted in‐plane under ultrasound guidance until it contacted the transverse process of T1. Then, 5 mL of 0.25% bupivacaine and 10 mL of 1.3% liposomal bupivacaine were injected into the fascial plane between the erector spinae muscle and the thoracic transverse process. This process was repeated on the contralateral side.

For the surgical procedure, the patient received general anesthesia through an endotracheal tube, maintained with propofol and remifentanil, to facilitate intraoperative neuromonitoring of motor evoked potentials, somatosensory evoked potentials, and electromyography. In addition to the procedural sedation, he was administered a total of 100 mcg of intravenous fentanyl during the intraoperative period. The surgery was performed via a posterior approach, with partial laminectomies at C3 and C7, C4‐6 laminoplasties, and right‐sided foraminotomies at C4‐5, C5‐6, and C6‐7. The surgery lasted for 3 h, with an estimated blood loss of 200 mL. 40 mL of 0.25% bupivacaine was infiltrated at the incision site by the surgeon before wound closure.

In the postanesthesia care unit (PACU), his highest numerical pain score was 7 out of 10, for which he received 75 mcg of intravenous fentanyl. After 39 min, he met the discharge criteria from the postanesthesia recovery unit and was then transferred to the orthopedic inpatient floor.

On postoperative day one, his highest pain score was 5 out of 10. The postoperative analgesia regimen included 5 or 10 mg of oral oxycodone as needed every four hours, 100 mg of oral tramadol as needed every four hours, and 1000 mg of oral acetaminophen scheduled every six hours. He required the as‐needed medications less frequently than their prescribed availability, taking tramadol twice daily and oxycodone on five occasions over the course of his admission. He met the discharge criteria on postoperative day one and was discharged home.

## 3. Discussion

This patient required no further intravenous opioid administration following their PACU stay and was able to receive minimal oral opioids while inpatient and upon discharge. His total oral morphine equivalents during hospitalization were 160 including preoperative and intraoperative doses of fentanyl, and he was discharged home on postoperative day one. No follow‐up occurred regarding pain scores or satisfaction postdischarge.

The present study adds to a growing body of literature in support of the use of the ESPB as an adjunct for perioperative analgesia in cervical spine surgery. Recent studies have demonstrated that ESPB can significantly reduce postoperative pain scores and opioid consumption when compared to multimodal analgesia alone. ESPB has been associated with improved satisfaction, earlier mobilization, and reduction in opioid‐related adverse events (specifically postoperative nausea and vomiting) [4].

ESPB are an effective regional anesthetic technique used to target the thoracic and abdominal wall. When compared to epidural and paravertebral blocks, ESPBs are less invasive and carry a lower risk of neurovascular or pleural injury. They have been successfully used to manage perioperative pain in various surgical procedures, including those involving the breast, thorax, abdomen, and thoracolumbar spine. Additionally, ESPBs have shown efficacy in treating chronic thoracic and shoulder pain [5].

The technique involves depositing local anesthetic in the plane between the transverse processes and the erector spinae muscles. Cadaveric studies suggest that this approach can facilitate the spread of local anesthetic into the paravertebral space, providing analgesia through the ventral and dorsal rami of spinal nerves. Clinically, this leads to somatic and visceral analgesia corresponding to the anesthetized spinal nerve distributions. The dorsal rami innervate the skin and posterior muscles that are typically surgically manipulated during posterior spinal surgeries. As a result, the local anesthetic’s spread to the dorsal rami contributes to the analgesia observed when ESPBs are applied in spinal and back surgery [6, 7]. A potential disadvantage of performing ESPBs near the cervical spine is the involvement of the phrenic nerve. In cadaveric investigations of ESPBs at the C6 and C7 levels, there was minimal staining of the phrenic nerve observed in a limited number of cadavers [8]. Therefore, the potential for phrenic nerve involvement should be carefully considered in patients with relevant respiratory comorbidities.

Previous research on ESPB blocks in posterior cervical spine surgery used 15 mL of 0.25% bupivacaine and dexamethasone bilaterally at T1 and demonstrated favorable results such as lower opioid use, reduced blood loss, quicker recovery, and increased patient satisfaction in comparison to standard multimodal analgesia alone [4]. A similar technique that included clonidine was reported in a patient undergoing anterior and posterior cervical spine instrumentation surgery, and it also resulted in similar favorable outcomes [9]. The administration of dexamethasone and clonidine in conjunction with local anesthetics has the potential to extend their duration of action up to 24 h, thereby yielding a lengthened analgesic benefit for the patient. Other methods of extending the duration of analgesia include insertion of a peripheral nerve catheter, but even this option comes with risks of infection, leaking, catheter migration, and dislodgement.

The use of liposomal bupivacaine as an adjunct in regional anesthesia has been studied in spine surgery. Liposomal bupivacaine is a sustained‐release multivesicular formulation of bupivacaine. After injection, bupivacaine is released from multivesicular liposomes and can provide extended analgesia up to 72 h, evidenced by decreased pain scores when compared with long‐acting local anesthetics [10]. The use of liposomal bupivacaine for ESPB blocks is currently an off‐label use, and further research is required to assess whether liposomal bupivacaine could be a viable option for use in ESPB blocks [11]. Currently, meta‐analyses done by Nguyen et al. and Daher et al. suggest liposomal bupivacaine may reduce postoperative pain scores, opioid consumption, and hospital length of stay when compared to traditional local anesthetics [12, 13]. The use of liposomal bupivacaine as described in this case may offer prolonged analgesia and further the opioid‐sparing effects seen with ESPB. This could be particularly relevant in patients who are at risk for opioid‐related complications. Furthermore, improving pain scores, reducing opioid consumption, and improving time to mobilization can help facilitate earlier hospital discharge which can assist in bed capacity issues in hospital facilities.

In conclusion, regional anesthesia for spine surgery is a new and evolving field. The ESPB shows promising benefits to patients undergoing cervical, thoracic, and lumbar spine surgeries. This case illustrates the potential benefits of using liposomal bupivacaine in ESPBs within a multimodal analgesic strategy for spine surgery. Ongoing research is warranted to define optimal strategies for using liposomal bupivacaine in ESBP.

## Consent

Informed consent and written permission were obtained from the patient to use and publish this case report. This case report was deemed Internal Review Board‐exempt by the Mayo Clinic Institutional Review Board. All protected health information has either been removed or de‐identified.

## Conflicts of Interest

The authors declare no conflicts of interest.

## Author Contributions

Hali Peterson and Mitchell Kerfeld participated in the design, execution, and analysis of the work presented, attest to the accuracy and validity of the contents, and have read and approved the final manuscript.

## Funding

No funding was used in this study.

## Data Availability

Data sharing is not applicable to this article as no datasets were generated or analyzed during the current study.
